# Research on the soil fractal characteristics and their correlation with soil properties in various forest types: insights from sub-humid area in Northern China

**DOI:** 10.1038/s41598-025-92215-1

**Published:** 2025-04-01

**Authors:** Yige Wang, Xiangyang Sun, Suyan Li, Bin Wei

**Affiliations:** 1 College of Forestry, Key Laboratory for Silviculture and Conservation of Ministry of Education, Beijing Forestry University, Beijing, 100083 China; 2 Liaoning Provincial Forestry Development Service Center, Liaoning Forestry and Grassland Administration, Shenyang, 110001 China

**Keywords:** Soil particle-size distribution, Fractal dimension, Soil physicochemical properties, Litter properties, Mountainous areas of Eastern Liaoning, Forest ecology, Forestry

## Abstract

Soil particle-size distribution (PSD) is one of the most important physical attributes due to its great influence on soil properties related to soil management and degradation. Thus, characterizing variations in the PSDs of soil are a major issue in environmental research. To date, the fractal model could well characterize PSD. Furthermore, scientific understanding and evaluation of forest soil quality is the basis for guiding ecological restoration and improvement of forest soil of degraded stands and select suitable tree species for afforestation purposes. Therefore, in this research the typical forest types: *Pinus koraiensis*, *Pinus sylvestris* var. *mongholica*, *Quercus mongolica*, *Juglans mandshurica* and mixed conifer-broadleaf (*Pinus koraiensis* × *Quercus mongolica*) forests in the mountains of eastern Liaoning were taken as the study objects. The topsoil (0–20 cm) and sub-topsoil (20–40 cm) samples, and litter were collected, and the relationship between the soil physiochemical properties and particle size characteristics under natural cultivation measures were evaluated and compared. The results indicated that the soil layer composition of forests were mainly sand (> 40%), followed by silt (> 25%) and clay (> 15%). The particles size characteristics showed well sorted (< 0.35), positive skewness (> 0.80) and narrow kurtosis state (1.11–1.61), and the singular fractal dimension (D) of soil was between 1.82 and 2.75. The mean particle size, D, litter and soil properties in forests were higher than those in non-forest cover control plots, and the Ds showed an increasing trend from conifer to broadleaf forests and from pure forest of single species to mixed conifer-broadleaf forests, and the recovery effect of topsoil soil was better. Pearson correlation analysis indicated that there is a positive correlation between physical and chemical indicators, and the singular fractal dimension and capacity dimension are significantly positively correlated with various indicators. Meanwhile, the multifractal dimensions are displayed as capacity dimension > correlation dimension > information dimension, indicating that the PSD is not completely ideal and uniform, thus it is still necessary to use the D to evaluate soil quality in combination with multifractal analysis. In conclusion, D is a sensitive and useful index because it quantifies changes in soil properties and it is highly recommended that broadleaf and mixed conifer-broadleaf forests are suitable for local afforestation for soil restoration purpose. Our results could provide a reliable scientific treatment method for forestry management and reconstruction in sub-humid area in Northern China and the same climate regions around the world.

## Introduction

The soil particle-size distribution (PSD) is one of the most significant physical attributes of soil and is the result of several complex geological, chemical, and biological processes^1,2^. It greatly influences soil hydraulic properties such as water retention characteristics, saturated hydraulic conductivities, soil bulk density, capillary porosity, et al.^3–5^. The characteristics of PSD have been found to be an ideal indicator of changes in soil structure, which can be influenced by management practices and degradation^6,7^. Therefore, characterizing variations in soil PSD is an important issue in environmental research and has garnered attention from many researchers^4,5,8–10^. Numerous studies have developed mathematical models for estimating soil PSD, with recent developments focusing on the use of fractal geometry as a descriptive tool for physical systems^11,12^. The common feature of fractal measures is their ability to characterize PSDs with parameters that retain the most information^13,14^. Laser diffraction, for instance, is a useful technique for measuring soil singular (volume) fractal dimension (D), and it is a reliable method for estimating PSD. Additionally, it helps in better understanding soil-forming processes and soil system performance^15,16^. Fractal theory and parameters have been applied to soil systems and are crucial in understanding and quantifying soil degradation and dynamics^17^. For example, Perfect and Kay (1991) developed a method to use fractal theory to characterize soil structure, while Tyler and Wheatcraft (1992) developed a mass-based distribution to estimate D of the PSD and determine the limits of fractal behavior and applications for soil PSD^5,18^. Rasiah et al. (1993) observed an inverse relationship between fractal dimension and organic matter content, while Pachepsky et al. (1995) found that simulated soil degradation caused an increase in Ds in one or more intervals of fractal behavior^19,20^. Fractal analysis has been shown to be sensitive to the coarsening process of soils during desertification^6,7^. Recent studies on the fractal nature of soils with various textures have shown a significant positive correlation between D of PSD and clay content, following a linear trend^9,21^, on n the other hand, Ds show a significant linear negative correlation with sand content^26,27^. Therefore, Ds of PSD are useful parameters for monitoring soil degradation and estimating the degree of soil desertification^8^. Furthermore, multifractal refers to a mathematical concept that describes complex structures with self-similarity^6–8^. Fractals exhibit similar characteristics at different scales, and multifractals exhibit self-similar structures at multiple levels of scale^22^. While most applications of fractal theory in soil science use a singular fractal approach, assuming that soil spatial distribution can be uniquely characterized by a single fractal dimension, it is unlikely that singular fractal distributions occur in landscapes due to the multiple processes that have shaped contemporary patterns^23^. Therefore, a singular fractal dimension may not always be sufficient to represent complex soil properties and the heterogeneous behavior of soil spatial variations^23^. A combination of all the fractal sets produces a multifractal spectrum that characterizes the variability and heterogeneity of the studied variable^6,7,23^. The advantage of the multifractal approach is that the multifractal parameters can be independent of the size of the studied objects, and no assumptions about the data following any specific distribution are required^24^. Potential applications of multifractal concepts include sampling designs, quantitative descriptions and comparisons of the studied properties, and the determination of processes influencing spatial distributions, among others^25^. Therefore, analyzing the PSD of soil should involve the use of both singular and multifractal methods.

The eastern mountainous area of Liaoning Province is the second-largest peninsula in China. The entire peninsula extends in a northeast-southwest direction, spanning a length of 340 km from the Lianshan Pass in the north to the Laotieshan Cape in the south, it has a width of 150 km in the north and covers an area of 2.94×10^4^ km^2,26,27^. The forest area in the Liaodong mountainous region covers 375.27×10^4^ ha, accounting for 67.34% of the total land area in the region, with a forest coverage rate of 61.46%^28^. The eastern mountainous area of Liaoning Province is a typical sub-humid area, which receives sufficient natural precipitation, which contributes to intact vegetation, abundant species, high soil nutrient content, and a well-functioning ecosystem that promotes plant growth^29,30^. However, with the regional and same climate regions worldwide, climate change, there has been an increase in temperatures and a decrease in rainfall, resulting in higher soil water evaporation and reduced effective water storage capacity^26,28,29^. As a result, the soil’s natural conditions have been disrupted, leading to a loss of support for normal tree growth and adversely affecting the growth quality of the forest stands, resulting in withering and even death of trees^31–34^. Even within the same region, the differences in tree characteristics and internal water-thermal conditions among different forest types directly affect soil water infiltration, organic matter humification and mineralization processes^20^. This, in turn, affects changes in soil physical properties, spatial distribution of organic matter, and nutrient contents^20^. Such as the typical forest stand types like *Pinus koraiensis*, *Pinus sylvestris* var. *mongolica*, *Quercus mongolica*, *Juglans mandshurica*), and mixed conifer-broadleaf (*Pinus koraiensis* × *Quercus mongolica*), nowadays difficulties such as slow growth, withered tops, and abnormal development, affected plantings that were made before the late 1990’s^26^. The problems raised concerns about the management of these forest stands. Studies have demonstrated that the main reasons for failure of stands were habitat changes, physical structures of soil, and loss of soil nutrients^26,28^. Currently, research in this area is not sufficiently thorough, particularly in the mountainous areas of eastern Liaoning Province. There is still a lack of comprehensive studies on the relationship between PSD characteristics and soil physicochemical properties in the understory of major forest stand types like *Pinus koraiensis*, *Pinus sylvestris* var. *mongolica*, *Quercus mongolica*, *Juglans mandshurica*), and mixed conifer-broadleaf (*Pinus koraiensis* × *Quercus mongolica*) using the commonly employed laser particle size analysis method.

Therefore, this study aims to focus on the current status and characteristics of secondary forests in the eastern mountainous area of Liaoning Province, with the goal of improving forest quality accurately and suitable afforestation tree species selections. Representative forest stands in this region, including *P. koraiensis*, *P. sylvestris*, *Q. mongolica*, *J. mandshurica*), and mixed conifer-broadleaf (*P. koraiensis* × *Q. mongolica*) were selected for comparative studies. Our specific goals were as follows: (i) to investigate the soil particle size composition, mean particle size, standard deviation, skewness, kurtosis, singular and multifractal dimensions, as well as the spatial distribution status of litter, soil physio-chemical properties, and (ii) their relationship with singular and multifractal dimensions under natural conditions. Based on the previous researches, the broadleaf and mixed conifer-broadleaf forests are superior to the coniferous and pure forest of single species for multiple functions and benefits^6,13,15,16,21^, and analyzing the soil PSD should involve the use of both singular and multifractal methods. Therefore, in this study, we fuehrer hypothesized that (1) the broadleaf and mixed conifer-broadleaf forests are suitable for local afforestation for soil restoration purposes, and timely restore, establish, and improve soil quality (fractal characteristics and physiochemical properties of forest soil) and (2) Evaluation of soil quality, it is better to use the singular fractal dimension in combination with multifractal analysis. The ultimate goal is to provide a theoretical basis for soil fertility control and regulation during forest management, maintain soil productivity in the understory, prevent forest degradation in the sub-humid area of north China. Furthermore, this study may improve the design and management of afforestation by using coniferous and broad-leaved forests in sub-humid area not only on the surveyed places, but also in the same climate zone worldwide that increase soil nutrients and improve the physical structure of soil, these changes would also be beneficial to stand development.

## Materials and methods

### Experiment site description

**Fig. 1:**
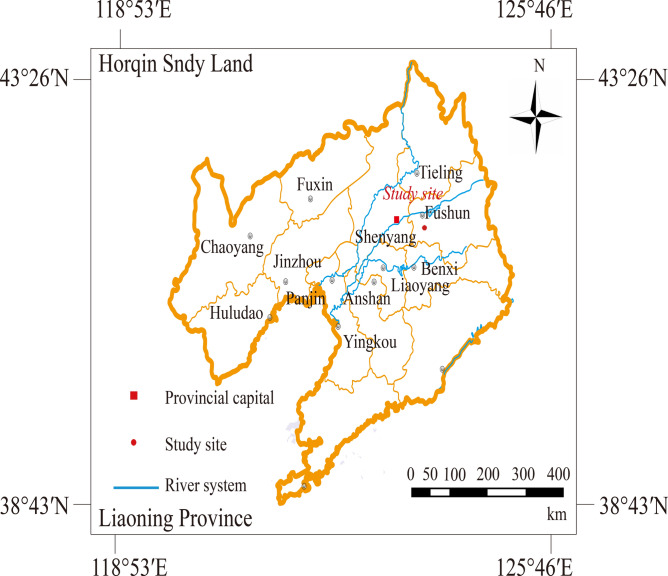
Geographical position of the study area. Map was generated using ArcGIS 10.0.0, 1:16, 000, 000 scale map of P.R. China was obtained from the National administration of surveying, mapping and geoinformation (http://bzdt.nasg.gov.cn/).

The study area is located in the forest of Qingyuan County, Fushun city. Fushun is a prefecture-level city in Liaoning Province and is one of the important industrial bases in the province. It is also the sub-center city of the Shenyang (provincial capital) economic zone. Geographically, Fushun city is located in the northeastern part of Liaoning Province. The city is divided into 4 districts and 3 counties, with a total area of 112.71 km^2^. As of the end of 2022, the registered population was 2.08 million people (see in Fig. 1).

Fushun is located in the middle latitude zone and has a typical monsoon climate, in winter, it is situated between the Mongolian high-pressure system and the Aleutian low-pressure system, with the north branch of the jet stream close to the upper atmosphere, which often brings northerly or westerly winds resulting in low temperatures; In spring, it is influenced by cyclones from the west, with increasing precipitation, additionally, due to the eastward movement of the upper trough blocked by Baikal lake, cold air from the north moves southward, causing late spring cold weather in the region; In summer, the area is mostly affected by southwestern or southeastern winds, with hot and rainy weather, occasionally, it is influenced by typhoons moving northward, causing heavy rainfall once a year; In autumn, it experiences early frost in late September under the influence of Siberian cold air^27^.

The forest of Qingyuan County, Fushun belongs to the Changbai vegetation region and also has the rich North China plant communities^27^. It has a relatively rich variety of plants, including 43 families, 95 genera, and 266 species of woody plants, and 90 families, 35 genera, and 712 species of wild herbaceous plants. The main representative tree species include *Pinus koraiensis* Sieb., *Abies holophylla* Maxim., *Abies fabri* (Mast.) Craib, *Larix olgensis* Henry., *Juglans mandshurica*, *Tilia amurensis* Rupr., *Fraxinus mandshurica* Rupr., *Populus davidiana* Dode., *Betula platyphylla* Suk., *Betula alleghaniensis*, and *Acer pictum* Thunb. Representative shrub species include *Pistacia vera* L., *Lespedeza bicolor* Turcz., *Acer pseudosieboldianum* (Pax) Komarov., *Acanthopanax senticosus* (Rupr. Maxim.) Harms., and *Lonicera ruprechtiana* Regel. Representative herbaceous species include *Dryopteris crassirhizoma* Nakai, *Brachybotrys paridiformis* Maxim. ex Oliv., and *Equisetum hyemale* L^27^. The main vegetation types include *Larix olgensis*, *Larix kaempferi*, *Quercus mongolica*, mixed forest of pine and broad-leaved trees, *Quercus wutaishansea*, *etc*. The research site is located in the forest of Qingyuan County, Fushun city in Liaoning Province (41°45’21" N- 41°42’13" N, 124°13’04" E- 124°17’07" E). The average elevation is 80 m above sea level. It is located in the temperate zone with a continental monsoon climate and distinct four seasons. Qingyuan County is typical sub-humid area in Northern China. The annual precipitation in this area averages 780 mm, with an annual evaporation averages 1500 mm. The effective accumulated temperature (≥5℃) is between 2620℃ and 3300℃, the sunshine duration is 150 d, the annual accumulated temperature is 2700–3200℃, the average annual temperature is in the range of 5–8℃ the average annual snowfall is 24 d, and the ground freeze depth is 1.2–1.4 m, the frost-free period lasts for 120 to 150 d. The main soil types are brown forest soil and dark brown forest soil^26,27^.

### Experimental design

Sample plots investigation. The selected research sites have similar forest age, slope direction, and position, representing various forest types. These include *P. koraiensis*, *P*. *mongholica*, *Q. mongolica*, *J. mandshurica*, and mixed conifer-broadleaf forest (with a ratio of 2:8 between *P. koraiensis* × *Q. mongolica*). In the year of 2023, a total of 40 sample plots (20 m × 20 m) were surveyed in detail, including 8 plots each for *P*. *koraiensis*, *P*. *sylvestris*, *Q*. *mongolica*, *J*. *mandshurica* and mixed conifer-broadleaf forest (8 sample plots were taken as reduplicates for each forest stand). The survey routes did not cross any rivers or roads. Starting from the center point of each plot, all standing trees with a diameter at breast height (DBH) greater than 5 cm were measured, and tree cores were extracted using an increment borer to determine the forest age. Meanwhile, 6 sample plots (20 m × 20 m) were selected in areas with herb cover density less than 20% as non-forest cover control plots (CK). Table 1 provides basic information about the sample plots, and all data are presented as mean values. The herb cover densities for the 5 forest types,* P*. *koraiensis*, *P*. *sylvestris*, *Q*. *mongolica*, *J*. *mandshurica*, and *P*. *koraiensis* × *Q*. *mongolica* forests, are 45, 35, 45, 40, and 65%, respectively.

**Table 1:** General characteristics of the surveyed forest plots^1^.

Forest type	A/m	*S*_d_	*S*_g_/°	*S*_p_	DBH/cm	H/m	*F*_a_/a	*H*_v_/%
*Pinus koraiensis*	647.0	W	17	Low	9.1	6.3	15	45
*Pinus sylvestris* var. *mongholica*	645.0	W	16	Low	7.3	5.1	16	35
*Quercus mongolica*	652.0	W	17	Low	13.1	10.1	15	45
*Juglans mandshurica*	646.0	SW	18	Low	10.1	10.3	16	40
mixed conifer-broadleaf forest	643.0	W	19	Low	10.0	10.4	18	65

^1^A presents the altitude, *S*_d_ is the slope direction, *S*_g_ presents the slope gradient, *S*_p_ is the slope position, *DBH* presents the diameter at breast height, *H* presents the tree height, *F*_a_ is the forest age, and *H*_v_ presents the herb coverage.

Investigation of litter properties. In each surveyed plot, 5 small plots (as reduplicates) measuring 0.2 m× 0.2 m were selected at random (not taken from the plot edge). All data were homogenized. Within each plot, litter, undecomposed layer (L), intermediate decomposed layer (F), and decomposed layer (H) were collected. Field observations were conducted to record relevant data such as litter layers and their thickness. The collected litter samples were then brought back to the laboratory and weighed using a portable electronic balance with a precision of 0.01%. Afterward, the samples were dried at 102℃ until constant weight, then the litter properties such as natural water holding capacity, maximum water holding rate, maximum water holding capacity, effective water holding rate, effective water holding capacity, effective retention capacity, and natural moisture content can be measured and calculated^35,36^.

Methods for Soil Property Analysis. All the soil samples (topsoil 0–20 cm and sub-topsoil 20–40 cm) were dried naturally in the laboratory for 2 d. All plant stems, roots, and tiny gravels were carefully removed, and then parts of the air-dried soil samples were hand sieved through 2 mm and 0.25 mm screens prior to laboratory analysis^37^. The soil physical properties were analyzed through the following methods: (1) Soil bulk density (bd), capillary porosity (*C*_P_), saturated soil moisture content (SMC) and Capillary moisture capacity (CMC) were measured through introduction of ring sampler; (2) total porosity (*T*_P_) was calculated using Eq. (1):

1 $${\text{TP}} = (1 - \frac{\gamma s}{{\rho s}}) \times 100$$

where *T*_P_ is the total porosity (%), and *ρ*_s_ is soil particle density which is equal to 2.73 g.cm^−315^.

Soil chemical properties were analyzed through the following: micro-Kjeldahl’s method for soil total nitrogen (*N*_T_) ; Mo-Sb colorimetric method for soil total phosphorus (*P*_T_); Hydrofluoric and perchloric acid (HF-HCLO acid)-flame photometer method for soil total potassium (*K*_T_); Alkali diffusion method for available nitrogen (*N*_avi_); Sodium bicarbonate (NaHCO3) digestion-Mo-Sb colorimetric method for soil available phosphorus (*P*_avi_); Ammonium acetate digestion-flame photometer method for soil rapid available potassium (*K*_avi_), and potassium dichromate wet combustion method for soil organic carbon (SOC), and pH value of soil was measured by potentiometric method^8,15,38^.

Soil fractal model descriptions and measurements. To measure the soil particles and fractal characteristics, the unscreened air-dried soil samples were pretreated with hydrogen peroxide (H_2_O_2_) solution (30%, w.w^−1^) to eliminate organic matter. Then, the soil aggregates were dispersed by adding sodium hexametaphosphate (NaPO_3_) and sonicating the samples for 30 s^6,7^. The pretreated soil samples were then analyzed using Malvern MasterSizer 2000 (Malvern Inc. England, UK), which has a laser diffraction technique with measurement range of 0.02–2000 mm and margin of error of 2%. Each sample was measured five times and the mean values were then obtained. The analysis results of soil PSD were output using U.S. Soil Taxonomy as follows: 0–2, 2–50, 50–100, 100–250, 250–500, 500–1000, and 1000–2000 μm^9,39^ D of soil PSD was calculated based on the volume PSD as follows:

2 $$\frac{V(r < Ri)}{{VT}} = (\frac{Ri}{{R\max }})_{{}}^{3 - D}$$

where r is the soil particle size, *R*_i_ is the soil particle size of grade i, *R*_max_ is the maximum value of soil particle size, V(r<*R*_i_) is the volume of soil particle size less than *R*_i_, and *V*_T_ is the total volume of soil particles^15–17^.

The mean particle size (*d*_0_) is a parameter that reflects the average condition of particle size in soil materials. The mean particle size is positively correlated with the proportion of fine particles^15–17^:

3 $$d0 = \frac{1}{3}(\Phi_{16} 16 + \Phi_{50} + \Phi_{84} )$$

In Equation (3), *Φ*_16_, *Φ*_50_, and *Φ*_84_ represent the soil particle sizes corresponding to the cumulative volume fractions of 16, 50, and 84% respectively^40^.

The standard deviation (*σ*_0_), skewness (*S*_0_), and kurtosis (*K*_0_) are calculated as follows in Eq. (4), (5), and (6):

4 $$\sigma 0 = \frac{1}{4}(\Phi_{50} - \Phi_{16} ) + \frac{1}{6.6}(\Phi_{95} - \Phi_{5} )$$

5 $$S0 = \frac{{\Phi_{16} + \Phi_{84} - 2\Phi_{50} }}{{2(\Phi_{84} - \Phi_{16} )}} + \frac{{\Phi_{5} + \Phi_{95} - 2\Phi_{50} }}{{2(\Phi_{95} - \Phi_{5} )}}$$

6 $${\rm K}0 = \frac{{\Phi_{95} - \Phi_{5} }}{{2.44(\Phi_{75} - \Phi_{25} )}}$$

**Fig. 2:**
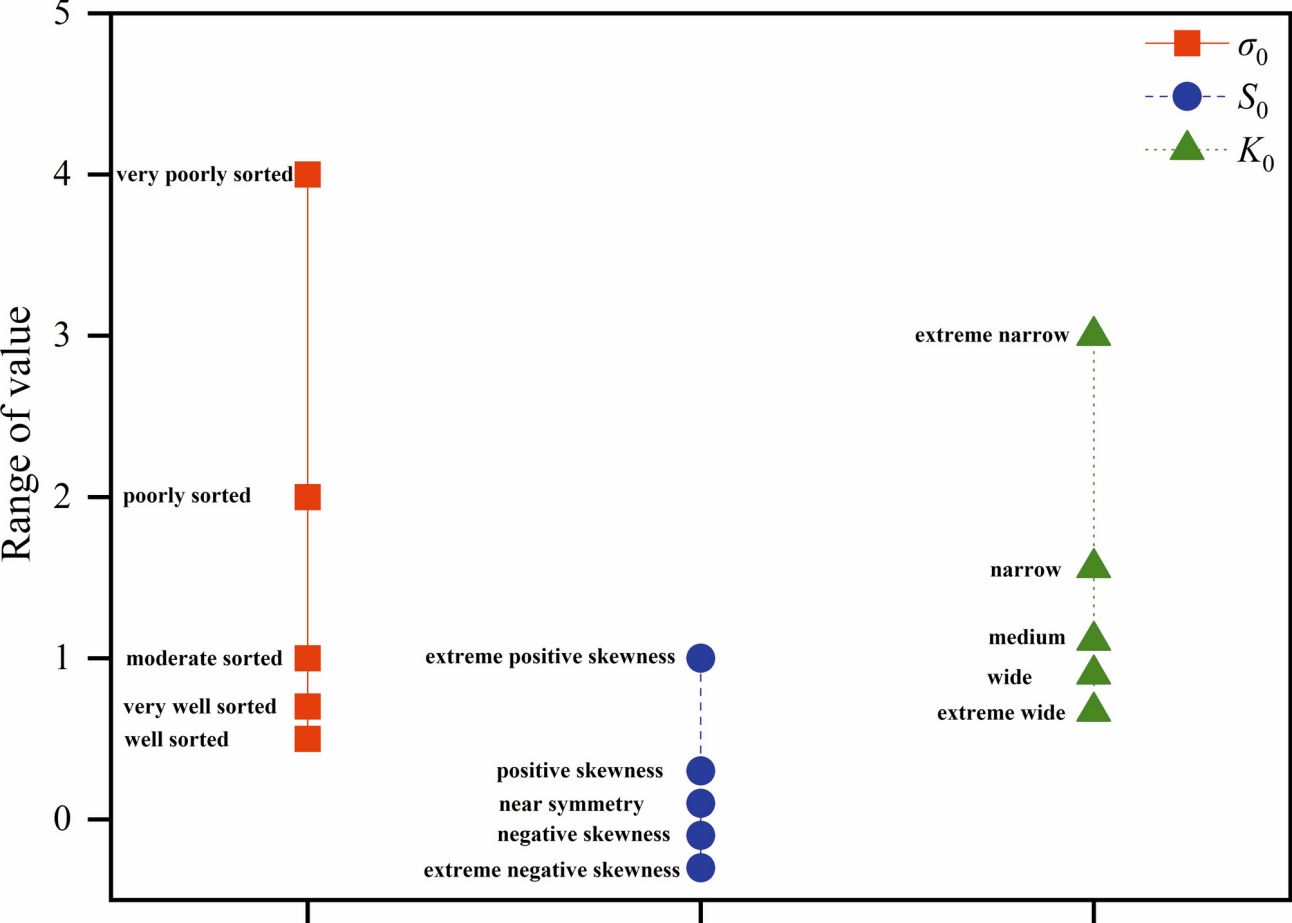
Description of the meaning of the standard deviation (*σ*_0_), skewness (*S*_0_), and kurtosis (*K*_0_) of D (Singular fractal dimension).

The descriptive meaning is shown in (Fig. 2):

To apply multifractal analysis methods and ensure that the lengths of each sub-interval in the particle size distribution are the same, a constant transformation is required for each sub-interval i within the measurement range of the laser particle size analyzer^10^. The calculation is as follows:

7 $$D\left( q \right) \approx \mathop {\lim }\limits_{\delta \to 0} \frac{1}{q - 1} \times \frac{{1g\left[ {\sum\limits_{i = 1}^{n\left( \delta \right)} {u_{i} \left( \delta \right)^{q} } } \right]}}{1g\delta }$$

8 $$D_{i} \approx \mathop {\lim }\limits_{\delta \to 0} \frac{{\sum\limits_{i = 1}^{n\left( \delta \right)} {u_{i} \left( \delta \right)1gu_{i} \left( \delta \right)} }}{1g\delta }q = 1$$

where *μ*_i(q, δ)_ represents the qth order probability of the ith sub-interval, where q is a real number. By using Eq. (7) and (8), we can calculate the multifractal parameters *D*_0_, *D*_1_, *D*_2_, and *D*_1_/*D*_0_ of PSD with a step size of 0.5 in the range of ~10≤q≤10. Among them, *D*_0_ is the capacity dimension when q=0, which is the classical fractal dimension used to measure the span of PSD. It provides the most basic information for studying PSD.* D*_1_ is the information dimension when q=1, which is related to Shannon diversity and can provide information about the irregularity of PSD. *D*_2_ is the correlation dimension when q=2, which is related to the Simpson diversity index and can provide information about the aggregation degree of soil particle distribution. *D*_1_/*D*_0_ is used to measure the heterogeneity of PSD^10^.

### Data processing and statistical analysis

Data were analyzed using SPSS software version 21.0 (IBM Inc. NC, USA). The differences in selected soil physicochemical properties and D (and related parameters), *D*_0_, *D*_1_, *D*_2_, and *D*_1_/*D*_0_ values among the forest types were compared using multiple comparison and one-way analysis of variance (ANOVA). A least-significant difference (LSD) test (at *P* < 0.05) was used to compare the means of soil variables. ANOVA with a LSD test was used with different letter in the same row are significantly different at the *P*=0.05. Pearson’s correlation coefficient and a two-tailed test were used to distinguish correlation (significantly correlated at *P* < 0.05 (0.05 level) and *P* < 0.01 (0.01 level)). Simple linear regression and correlation analysis were performed using OriginLab OriginPro 9.0 software (OriginLab Inc., Northampton, MA, USA) to identify the relationships between D, D0 and the selected soil properties (at the 0.05 level and 0.01 level). Data processing and plotting were also completed using SciDAVis (DHI Group, Inc., NY, USA).

## Results

### Soil particle size distribution

According to supplementary information file (Table S1), it can be observed that the soil particle size distribution in different forest stands is dominated by sand particles, accounting for over 40% of the composition. The next major fraction is silt, with a percentage above 25%. The clay content is the lowest, with an average volume percentage exceeding 15%. When comparing from pure tree species forests to mixed conifer-broadleaf forests, it is evident that the sand content gradually decreases while the clay and silt content show an increasing trend in all soil layers. In terms of individual forest types, the topsoil and sub-topsoil layers of *P*. *mongolia* forests have the highest sand content, measuring 56.1±4.2 and 58.4±3.1% respectively. While, the topsoil and sub-topsoil layers of *P*. *koraiensis* × *Q*. *mongolica* forests have the lowest sand content, measuring 43.7±4.2 and 41.4±2.9% respectively. Moreover, this trend of change is more pronounced in broadleaved forests compared to coniferous forests. In *Q*. *mongolica* forests, the clay and silt contents in the topsoil and sub-topsoil layers are 18.4±3.1% and 17.7±3.−%, 32.9±2.3% and 36.1±4.3%, respectively. Compared to the CK, the particle size distribution in the forest understories of various forest types also follows a similar pattern. The clay, silt, and sand contents in the topsoil and sub-topsoil layers of the CK are the highest, measuring 8.5±1.0 and 6.9±1.5%, 23.4±3.3 and 23.3±2.2%, 68.1±4.3 and 69.8±3.3%, respectively. Furthermore, the vertical distribution of soil particle size reveals that the sub-topsoil soil has a higher proportion of sand particles, while the top soil has relatively higher proportions of clay and silt particles.

### Singular and multifractal dimensions and their parameters

**Fig. 3:**
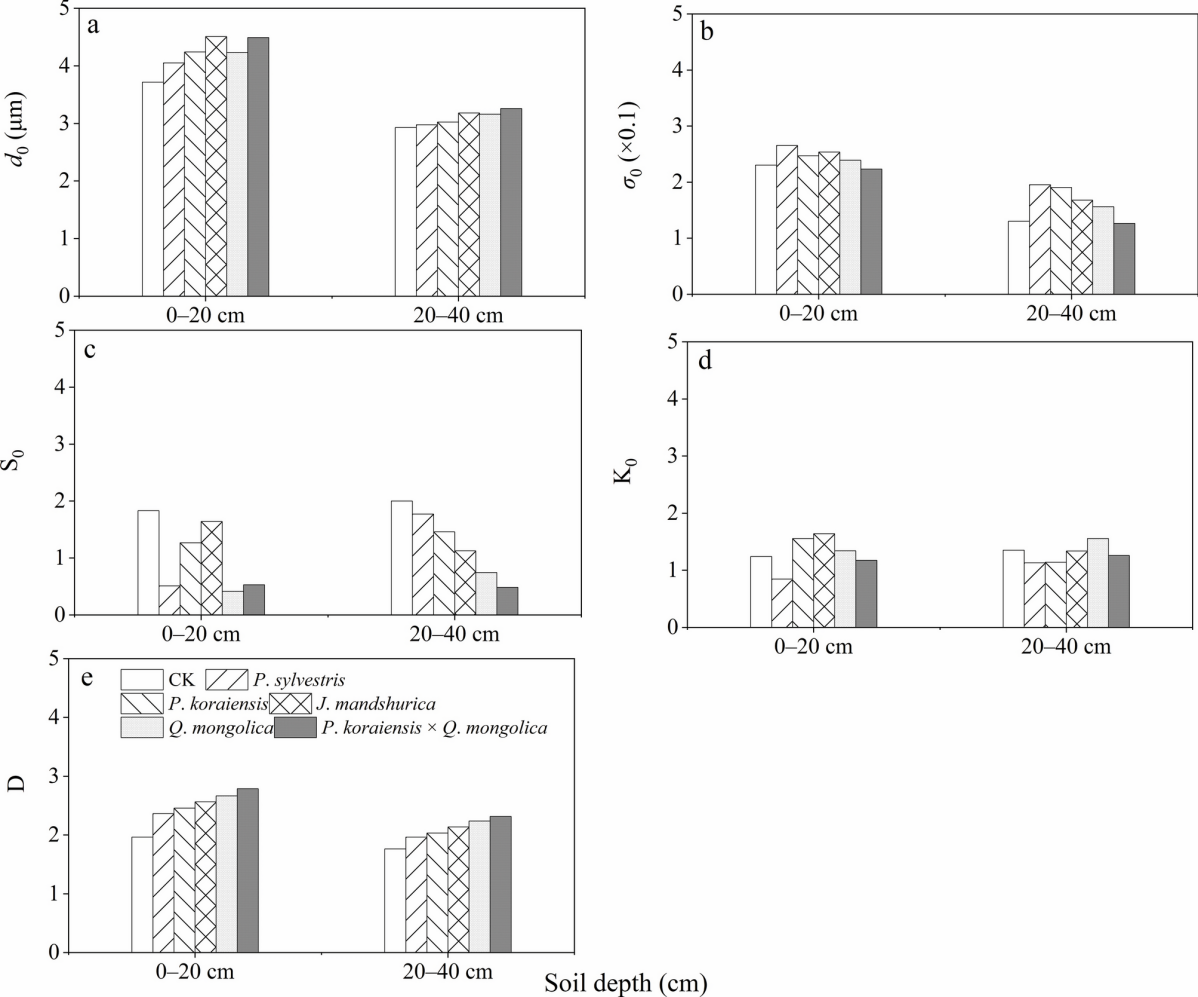
D and its parameters, the mean particle size *d*_0_, (**a**), *σ*_0_ (**b**), *S*_0_ (**c**), and *K*_0_ (**d**), and D (**e**).

The *d*_0_ of the soil in each layer of the forest in the study area is larger than that of the CK. The* d*_0_ in the topsoil and sub-topsoil layers range from *P*. *mongholica*, *P*. *koraiensis*, *J*. *mandshurica*, *Q*. *mongolica*, *P*. *koraiensis* × *Q*. *mongolica* to CK, which are 3.71, 4.03, 4.22, 4.50, 4.22, and 4.46 μm, and 2.91, 2.96, 3.01, 3.14, 3.13, and 3.23 μm, respectively (see Fig. 3a). As shown in Fig. 3b, the soil PSD in the soil of various forest types is relatively concentrated, indicating well sorted conditions (*σ*_0_< 0.35). Figure 3c showed that the *S*_0_ values of CK, *P*. *koraiensis*, and *J*. *mandshurica* forests are relatively large in the topsoil layer, while the *S*_0_ values of *P*. *koraiensis* × *Q*. *mongolica* forest, *Q*. *mongolica*, and *P*. *mongholica* forests are relatively small in the sub-topsoil. The *S*_0_ values of CK, *P*. *mongholica*, *P*. *koraiensis*, and *J*. *mandshurica* forests are relatively large, while the *S*_0_ values of *Q*. *mongolica* and *P*. *koraiensis* × *Q*. *mongolica* forests are relatively small. As shown in Fig. 3d, except for *P*. *mongholica* (0.83) and *J*. *mandshurica* (1.82) in the topsoil, and *Q*. *mongolica* forest (1.61) in the sub-topsoil, the *K*_0_ values of other forests are narrow (1.11<*K*_0_<1.56), and the *K*_0_ values of *P*. *koraiensis* × *Q*. *mongolica* forest are smaller than that of CK, indicating a concentrated soil PSD. Figure 3e shows that from pure single tree species forests to mixed conifer-broadleaf forest, from coniferous forests to broadleaf forests, and compared to the CK, the D values gradually increases. Moreover, the D values of the topsoil is higher than that of the sub-topsoil. The Ds in the topsoil and sub-topsoil layers range from *P*. *mongholica*, *P*. *koraiensis*, *J*. *mandshurica*, *Q*. *mongolica*, *P*. *koraiensis* × *Q*. *mongolica* forests to CK, which are 2.32, 2.45, 2.54, 2.63, 2.75, 1.93, and 1.94, 2.01, 2.12, 2.22, 2.31, and 1.82 respectively.

**Fig. 4:**
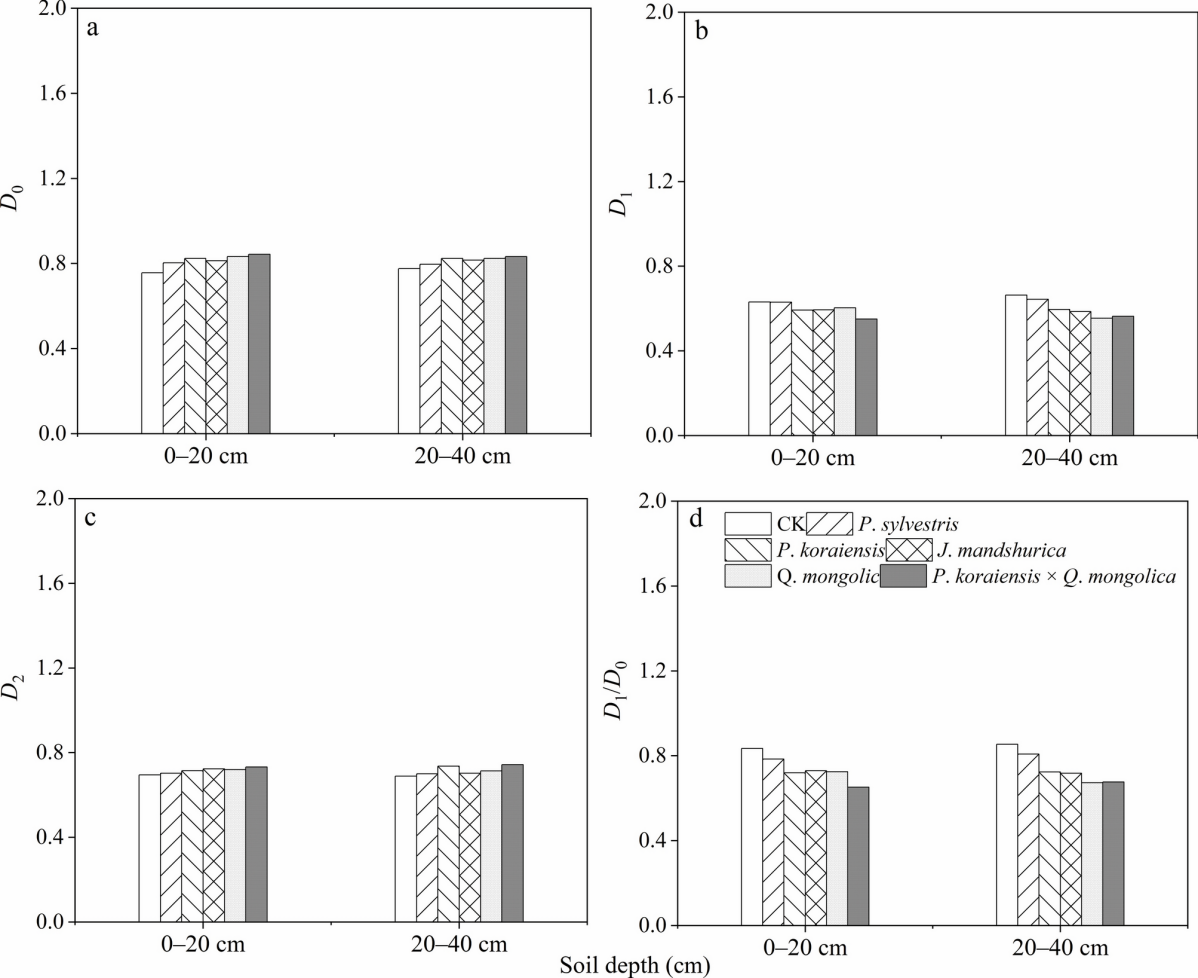
Multi-fractal dimensions, the capacity dimension (*D*_0_, **a**), information dimension (*D*_1_, **b**), correlation dimension (*D*_2_, **c**), and *D*_1_/*D*_0_ (**d**).

The values of the multifractal parameters *D*_0_, *D*_1_, *D*_2_, and *D*_1_/*D*_0_ can be obtained to analyze the characteristics of soil PSD. According to Fig. 4a, the *D*_0_ ranges from 0.75 to 0.85 in the topsoil and from 0.77 to 0.82 in the sub-topsoil. Among them, the highest values were observed in* P*. *koraiensis*, *Q*. *mongolica*, *J*. *mandshurica*, and *P*. *koraiensis* × *Q*. *mongolica* forests, followed by *P*. *sylvestris* forests, and the lowest values are observed in CK. A larger *D*_0_ value indicates a wider range of soil PSD, indicating that the PSD in *P*. koraiensis, *Q. mongolica*, *J. mandshurica*, and *P. koraiensis* × *Q. mongolica* forests is relatively wide, with particles distributed extensively in various size segments, while the PSD range in the *P*. *sylvestris* forests and CK is the narrowest. According to Fig. 4b, the *D*_1_ ranges from 0.54 to 0.62 in the topsoil and from 0.54 to 0.65 in the sub-topsoil; The highest value was observed in the CK, followed by *P*. *sylvestris* forests, and the lowest values were observed in *Q*. *mongolica* forests and *P*. *koraiensis* × *Q*. *mongolica* forests. A larger *D*_1_ value indicates a higher degree of irregularity in the distribution of soil particles; *P*. *sylvestris* forests and CK exhibit a higher degree of irregular distribution, while *Q*. *mongolica* forests and *P*. *koraiensis* × *Q*. *mongolica* forests show a more regular PSD. According to Fig. 4c, *D*_2_ ranges from 0.68 to 0.72 in the topsoil and from 0.67 to 0.73 in the sub-topsoil; The highest *D*_2_ value is observed in the *P*. *koraiensis* × *Q*. *mongolica* forests, followed by* Q*. *mongolica*, *J*. *mandshurica*, *P*. *koraiensis*, and *P*. *sylvestris* forests; The lowest *D*_2_ value is observed in the CK. A larger *D*_2_ value indicates a greater uniformity in the distribution of soil particles, but with more scattered distribution; The highest *D*_2_ value in the *P*. *koraiensis* × *Q*. *mongolica* forests indicates the highest level of uniformity in the distribution of soil particles, with a relatively dispersed particle distribution. According to Fig. 4d, the *D*_1_/*D*_0_ ranges from 0.64 to 0.81; The highest value is observed in the CK, followed by *P*. *sylvestris* forests, and the lowest *D*_1_/*D*_0_ value is observed in the mixed forests of conifers and broadleaf forests; A larger *D*_1_/*D*_0_ value indicates a higher degree of heterogeneity in the distribution of soil particles; The highest* D*_1_/*D*_0_ value in the CK indicates the highest level of heterogeneity in the distribution of soil particles. From the above analysis, it can be seen that the multifractal parameters follow certain regularities. This indicates that the CK has a wider range of soil particle size distribution and a greater degree of irregularity and heterogeneity in particle distribution. In contrast, the* P*. *koraiensis* × *Q*. *mongolica* forests have a narrower range of soil particle size distribution and a higher level of uniformity and regularity in particle distribution. Meanwhile, the multifractal parameters show the order of *D*_0_ > *D*_2_ > *D*_1_, indicating that when viewed from D, the PSD in each layer of the soil is relatively concentrated, and the sorting condition is excellent. However, the results of multifractal dimensions indicate that the soil PSD is not completely ideal and uniform. Therefore, it is still necessary to use multifractal analysis in combination with singular fractal dimensions to evaluate soil quality.

### Relationships between fractal dimensions and soil particle contents

**Fig. 5:**
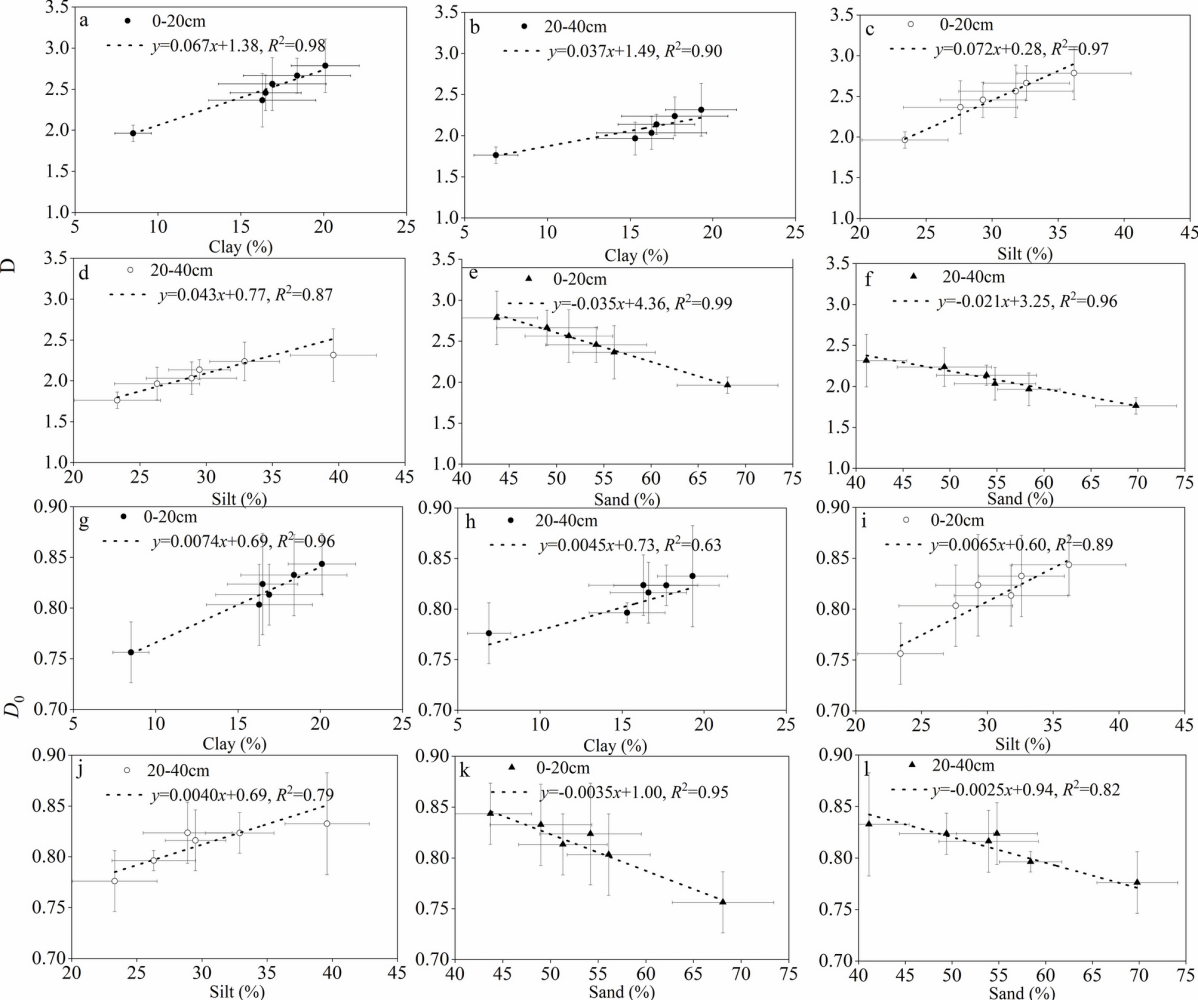
Relationships between D and *D*_0_ values and soil particle-size distribution (PSD) contents. Vertical and horizontal bars indicate standard errors of means. Linear regression analysis was applied and determination coefficients (*R*^2^) are also shown.

The relationship between soil particle size composition and soil singular and multi-fractal dimensions (D and *D*_0_) under various forest types can be observed from Figs. 3 and 4. The soil fractal dimensions of each soil layer are positively correlated with clay and silt content, while negatively correlated with sand content. Among them, the linear fitting coefficient of the topsoil is higher than that of the sub-topsoil. In the topsoil, the D has the best fitting effect with silt and sand, respectively, given by *y*=0.067*x*+1.38, *R*^2^=0.98 and *y*=−0.035*x*+4.36, *R*^2^=0.99 (Fig. 5a,e); the *D*_0_ has the best linear fitting effect with clay and sand, respectively, given by *y*=0.0074*x*+0.69, *R*^2^=0.96 and *y*=−0.0035*x*+1.00, *R*^2^=0.95 (Fig. 5g,k). The D exhibits a higher degree of fit with the soil particle size composition than the *D*_0_, and the linear equation fitting coefficient between the *D*_0_ and the soil particle size composition in the sub-topsoil is lower than that between the D and the soil particle size composition in the topsoil (Fig. 5b,d,f,h,j,l).

### Litter properties

The results of litter accumulation were shown in supplementary information file (Table S2). The thickness of the litter layer under different forests is as follows: *P*. *koraiensis*< *P*. *sylvestris*< *Q*. *mongolica*< *J*. *mandshurica*< *P*. *koraiensis* × *Q*. *mongolica*. The thickness of the L, F, and H layers was the largest in the *P*. *koraiensis* × *Q*. *mongolica* forest. The litter accumulation of the forest types is as follows: *P*. *koraiensis*< *P*. *sylvestris*< *Q*. *mongolica*< *J*. *mandshurica*< *P*. *koraiensis* × *Q*. *mongolica*. The accumulation of the L layer of litter in each forest type is more than 1.3 times that of the F layer, and the proportion of the L layer in the litter accumulation is the largest in the *P*. *koraiensis* × *Q*. *mongolica* forests, accounting for 43.36%. The results showed that the litter accumulation in the *J*. *mandshurica*, *Q*. *mongolica*, and *P*. *koraiensis* ×*Q*. *mongolica* forests are higher than that in the *P*. *koraiensis* and *P*. *sylvestris* forests, and the L layer and the F layer have similar characteristics.

As shown in supplementary information file (Table S3), the maximum water holding capacity of litter in *P*. *koraiensis* ×*Q*. *mongolica* forests is 20.81 t.hm^−2^, equivalent to a water depth of 2.08 mm. The maximum water holding capacity of litter in *P*. *koraiensis* forests is the smallest, at 14.93 t.hm^−2^, equivalent to a water depth of 1.49 mm. For all forest types, the maximum water holding capacity of the litter layer is highest in the L layer, followed by the F layer, and lowest in the H layer. Among them, the difference in maximum water holding capacity of litter layers is greatest in *P*. *koraiensis* ×*Q*. *mongolica* forests, where the maximum water holding capacity of the L layer is 2.09 times that of the H layer. The maximum water holding rate of litter is between 190.70 and 330.20%. *P*. *koraiensis* ×*Q*. *mongolica* forests have the highest effective water holding capacity, which is 21.32 t.hm^–2^, equivalent to a water depth of 2.13 mm. *P*. *koraiensis* forests have the lowest effective water holding capacity, which is 14.93 t.hm^–2^, equivalent to a water depth of 1.49 mm. In terms of effective retention capacity, the highest in *P*. *koraiensis* ×*Q*. *mongolica* forests, which is 19.97 t.hm^–2^. The lowest is in* Q*. *mongolica* forests, which is 10.27 t.hm^-2^. The natural water holding capacity, maximum water holding rate, maximum water holding capacity, effective water holding rate, effective water holding capacity, effective retention capacity, and natural moisture content of litter in forest types are in the same order of L layer > F layer > H layer.

### Soil physicochemical properties

**Fig. 6:**
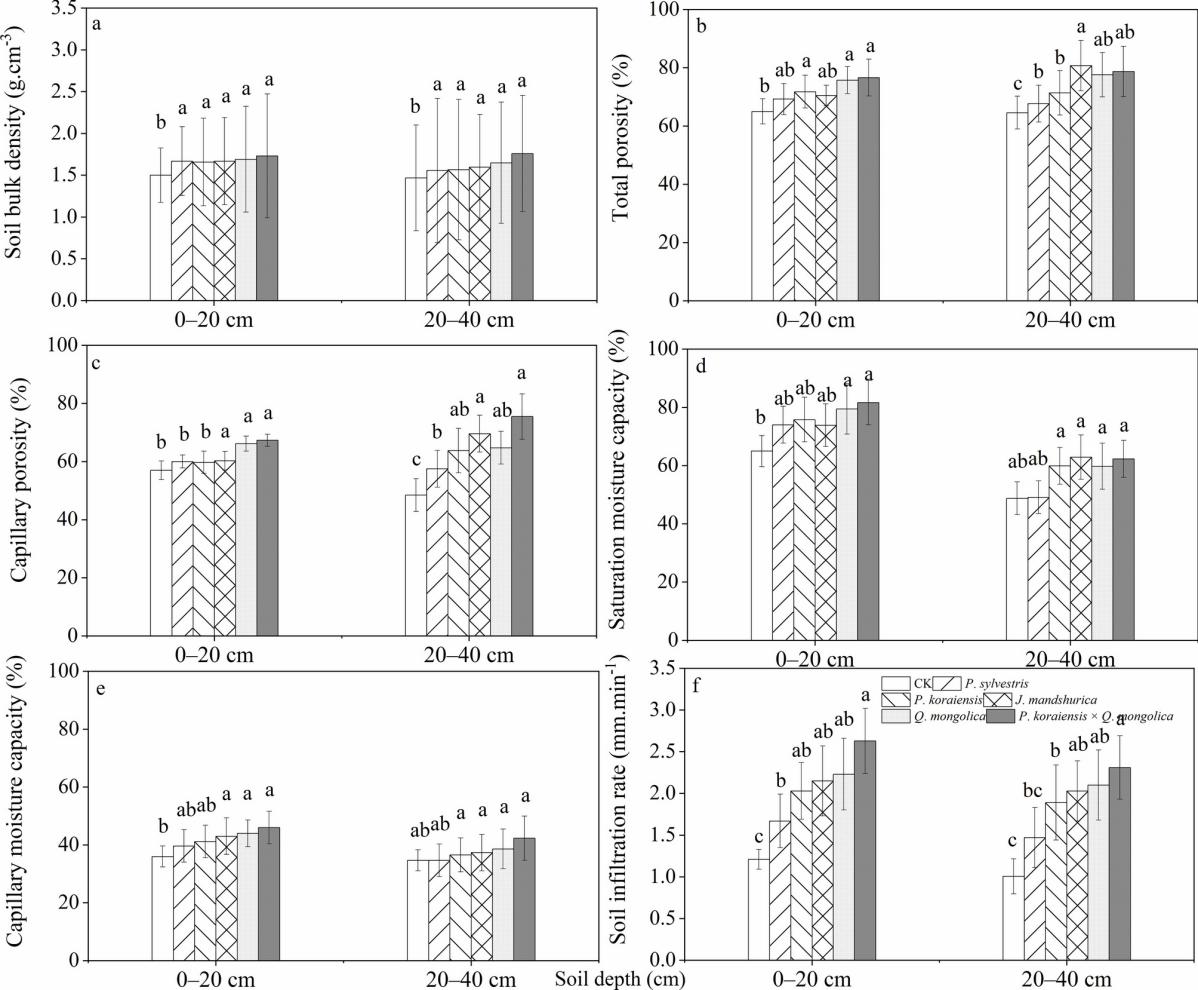
Variations of soil physical properties (soil bulk density (**a**), total porosity (**b**), capillary porosity (**c**), saturation moisture capacity and (**d**), capillary moisture capacity (**e**), and soil infiltration rate (**f**)) in various forest types. Vertical bars indicate standard errors of means. One-way analysis of variance with a least-significant difference test was used with different letter in the same row are significantly different at the *P* = 0.05. Same below.

Based on the comparison results of Fig. 6a,b, and e, there is no significant difference in soil bulk density, total porosity, and capillary moisture capacity among different forests (*P*>0.05), but there are significant differences compared to the CK (*P*<0.05). Figure 6c, d, and f showed that there are more significant differences in capillary porosity, saturation moisture capacity, soil infiltration rate between the CK and* P*. *sylvestris* and other forests (*P*<0.05). Overall, the soil physical indicators of *P*. *koraiensis*, *P*. *sylvestris*, *Q*. *mongolica*, *J*. *mandshurica*, *P*. *koraiensis* × *Q*. *mongolica* forests showed an increasing trend. Compared to coniferous forests, this trend is more pronounced in broad-leaved forests. However, the soil physical properties of the CK are at a lower level for all indicators. Meanwhile, the vertical distribution of the soil showed that the topsoil has significantly better physical properties than the sub-topsoil.

**Fig. 7:**
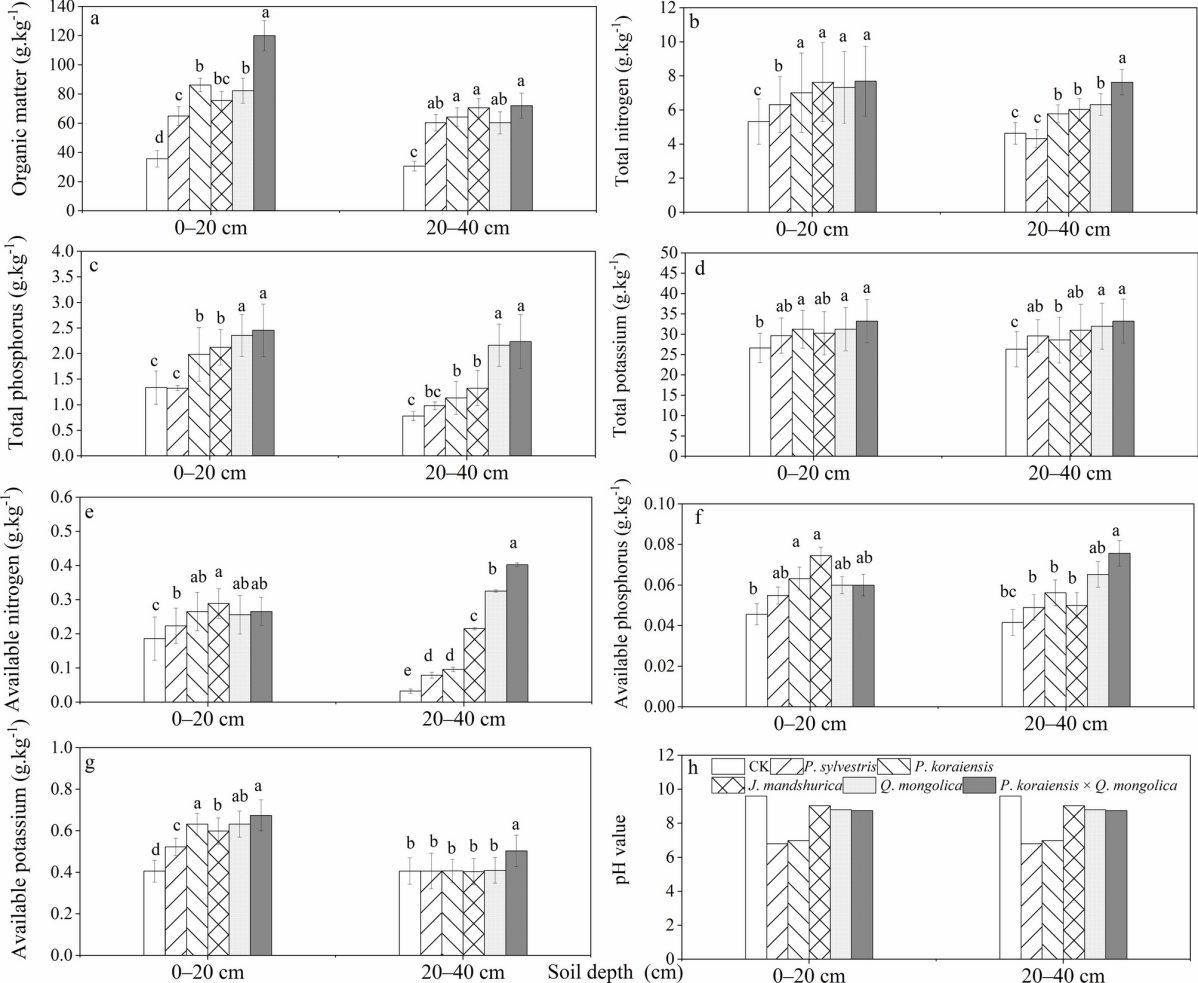
Variations of soil chemical properties (organic matter (**a**), total nitrogen (**b**), total phosphorus (**c**), total potassium (**d**), available nitrogen (**e**), available phosphorus (**f**), available potassium (**g**), and pH value (**h**)) in various forest types.

From Fig. 7, it can be seen that the change trend of soil chemical properties is relatively consistent with that of soil physical properties (compare to Fig. 6). In general, the soil chemical stoichiometric indicators of different layers in *P*. *koraiensis*, *P*. *sylvestris*, *Q*. *mongolica*, *J*. *mandshurica*, *P*. *koraiensis* × *Q*. *mongolica* forests showed an increasing trend. Compared to coniferous forests, the change trend is more pronounced in broad-leaved forests. Figure 7b,d,f showed no significant differences among different forest types (*P*>0.05), but there are significant differences compared to the CK(*P*<0.05). Figure 7a,c,e,g show that the differences between the CK and *P*. *sylvestris* forest and other forest types are more significant (*P*<0.05). The soil chemical stoichiometric indicators in the CK are generally at a lower level. Additionally, the topsoil chemical properties are significantly better than the sub-topsoil. It is worth mentioning that the pH values of the soils under *P*. *sylvestris* forest, CK, *P*. *koraiensis*, *J*. *mandshurica*, *Q*. *mongolica*, and *P*. *koraiensis* × *Q*. *mongolica* forests are 6.7, 9.5, 6.8, 9.1, 8.6, and 8.5, respectively. There are no significant differences in soil pH values among different locations, and there is no apparent acidification, indicating that soil stress is not severe, especially in the *P*. *koraiensis* and *P*. *sylvestris* forests.

### Relationship between fractal dimensions and soil physicochemical properties of soil subsections in different forest types

The results of the Pearson correlation analysis between singular and multifractal dimensions and soil physical property indicators were as follows: there is a strong correlation among the indicators. Specifically, the D and *D*_0_ showed significant positive correlations with various physical property indicators. While, the correlation *D*_1_ and *D*_1_/*D*_0_ showed significant negative correlations with the various physical property indicators. Additionally, we found a strong correlation among the soil physical property indicators themselves, with the soil capillary moisture capacity showing the strongest correlation with the various indicators (Table 2). This suggests that the soil capillary moisture capacity may be an important soil physical property, closely related to other indicators.

**Table 2:** Pearson correlation coefficients among fractal dimensions and soil physical properties^1^.

Indicator	Soil layer	D	*D*_0_	*D*_1_	*D*_2_	*D*_1_/*D*_0_	*T*_P_	*C*_P_	SMC	CMC
D	Topsoil	1	0.91*	−0.82*	0.92**	−0.91**	0.91**	0.82*	0.93*	0.96**
	Sub-topsoil	1	0.91**	−0.93**	0.65	−0.93**	0.84*	0.91*	0.92**	0.82*
*D*_0_	Topsoil	0.91**	1	−0.90**	0.84*	−0.91**	0.72*	0.72*	0.75**	0.90**
	Sub-topsoil	0.91**	1	−0.92**	0.82*	−0.93**	0.80*	0.81*	0.90**	0.91**
*D*_1_	Topsoil	−0.82*	−0.90**	1	−0.92**	0.94**	-0.75*	−0.72*	−0.73*	−0.84*
	Sub-topsoil	−0.93**	−0.92**	1	−0.63	0.92**	-0.81*	−0.91**	−0.83*	−0.91**
*D*_2_	Topsoil	0.92**	0.84*	−0.92**	1	−0.93**	0.83*	0.73*	0.81*	0.94**
	Sub-topsoil	0.65	0.82*	−0.63	1	−0.71	0.43	0.41	0.72	0.65
*D*_1_/*D*_0_	Topsoil	−0.91**	−0.91**	0.94**	−0.93**	1	−0.90**	−0.81*	−0.95**	−0.92**
	Sub-topsoil	−0.93**	−0.93**	0.92**	−0.71	1	−0.84*	−0.83*	−0.86*	−0.91**
*T*_P_	Topsoil	0.91**	0.72*	−0.75*	0.83*	−0.90**	1	0.92**	0.93**	0.93**
	Sub-topsoil	0.84*	0.80*	−0.81*	0.43	−0.84*	1	0.91**	0.81*	0.90**
*C*_P_	Topsoil	0.82*	0.72*	−0.72*	0.73*	−0.81*	0.92**	1	0.91**	0.82*
	Sub-topsoil	0.91*	0.81*	−0.91**	0.41	−0.83*	0.91**	1	0.91**	0.93**
SMC	Topsoil	0.93*	0.75*	−0.73*	0.81*	−0.95*	0.93**	0.91**	1	0.93**
	Sub-topsoil	0.92**	0.90**	−0.83*	0.72	−0.86*	0.81*	0.91**	1	0.91**
CMC	Topsoil	0.96**	0.90*	−0.84*	0.94**	−0.92**	0.93**	0.82*	0.93**	1
	Sub-topsoil	0.82*	0.91*	−0.91*	0.65	−0.91**	0.90**	0.93**	0.91*	1

^1^D presents the singular fractal dimension, topsoil is 0–20 cm depth of soil, sub-top soil is 20–40 cm depth of soil, *D*_0_ presents the capacity dimension, *D*_1_ presents information dimension, *D*_2_ presents correlation dimension, *T*_P_ presents total porosity, *C*_P_ presents capillary porosity, *SMC* presents saturation moisture capacity, *CMC* presents saturation moisture capacity. Same below.

**Table 3:** Pearson correlation coefficients among fractal dimensions and soil chemical properties^1^.

Indicator	Soil layer	D	*D*_0_	*D*_1_	*D*_2_	*D*_1_/*D*_0_	SOC	*N*_T_	*P*_T_	*K*_T_	*N*avi	*P*avi	*K*avi
D	Topsoil	1	0.91**	−0.81*	0.92**	−0.92**	0.91**	0.93**	0.85*	0.94**	0.82*	0.65	0.95**
	Sub-topsoil	1	0.91**	−0.93**	0.65	−0.93**	0.82*	0.85*	0.93**	0.92**	0.91**	0.91**	0.63
*D*_0_	Topsoil	0.91**	1	−0.92**	0.84*	−0.94**	0.83*	0.82*	0.74*	0.72*	0.74*	0.81*	0.54
	Sub-topsoil	0.91**	1	−0.92**	0.83*	−0.94**	0.84*	0.83*	0.74	0.83*	0.75	0.84*	0.52
*D*_1_	Topsoil	−0.81*	−0.92**	1	0.92**	0.93**	−0.92**	−0.81*	−0.83*	−0.83*	−0.70	−0.52	−0.85*
	Sub-topsoil	−0.93*	−0.92**	1	−0.64	0.92**	−0.71	−0.84*	−0.86*	−0.83*	−0.84*	−0.81*	−0.46
*D*_2_	Topsoil	0.92**	0.84*	0.92**	1	−0.94**	0.90*	0.93**	0.91**	0.91*	0.85*	0.71	0.91**
	Sub-topsoil	0.65	0.83*	−0.64	1	−0.72	0.64	0.74*	0.60	0.54	0.54	0.85*	0.64
*D*_1_/*D*_0_	Topsoil	−0.92**	−0.94**	0.93**	−0.94**	1	−0.94**	−0.94**	−0.95**	−0.92**	−0.81*	−0.60	−0.93**
	Sub-topsoil	−0.93**	−0.94**	0.92**	−0.72	1	−0.73*	−0.84*	−0.85*	−0.83*	−0.84*	−0.81*	−0.46
SOC	Topsoil	0.91**	0.83*	−0.92**	0.90**	−0.94**	1	0.83*	0.85*	0.97**	0.72	0.50	0.92**
	Sub-topsoil	0.82	0.84*	−0.71	0.64	-0.73	1	0.63	0.56	0.80*	0.62	0.62	0.26
*N*_T_	Topsoil	0.93**	0.82*	−0.81*	0.93**	−0.94**	0.83*	1	0.91**	0.84*	0.95**	0.81*	0.93**
	Sub-topsoil	0.85*	0.83*	−0.84*	0.74	−0.84*	0.63	1	0.85*	0.80*	0.91*	0.83*	0.71
*P*_T_	Topsoil	0.85*	0.74*	−0.83*	0.91**	−0.95**	0.85*	0.91**	1	0.82*	0.81	0.61	0.84*
	Sub-topsoil	0.93**	0.74*	−0.86*	0.60	−0.85*	0.56	0.85*	1	0.91**	0.94**	0.91**	0.64
*K*_T_	Topsoil	0.94**	0.72	−0.83*	0.91**	−0.92**	0.97**	0.84**	0.82*	1	0.73	0.52	0.94**
	Sub-topsoil	0.92**	0.83*	−0.83*	0.54	−0.83*	0.80*	0.80*	0.91**	1	0.91**	0.81*	0.60
*N*avi	Topsoil	0.82*	0.74*	−0.70	0.85*	−0.81*	0.72	0.95**	0.81	0.73	1	0.91**	0.83*
	Sub-topsoil	0.91**	0.75*	−0.84*	0.54	−0.84*	0.62	0.91**	0.94**	0.91**	1	0.92**	0.73
*P*avi	Topsoil	0.65	0.81*	−0.52	0.71	−0.60	0.50	0.81*	0.61	0.52	0.91**	1	0.64
	Sub-topsoil	0.91*	0.84*	−0.81*	0.85*	−0.81*	0.62	0.83*	0.91**	0.81*	0.92**	1	0.75*
*K*avi	Topsoil	0.95**	0.54	−0.85*	0.91**	−0.93**	0.92**	0.93**	0.84*	0.94**	0.83*	0.64	1
	Sub-topsoil	0.63	0.52	−0.46	0.64	−0.46	0.26	0.71	0.64	0.60	0.73	0.75	1

^1^*N*_T_ presents the total nitrogen, *P*_T_ presents the total phosphorus, *K*_T_ presents total potassium, *N*_avi_ presents available nitrogen, *P*_avi_ presents available phosphorus, *K*_avi_ presents available potassium.

The results of the Pearson correlation analysis between fractal dimensions and soil chemical properties are as follows: Consistent with the analysis results of soil physical properties, the D and *D*_0_ showed a significant positive correlation with various chemical property indicators. On the other hand, the *D*_1_ and *D*_1_/*D*_0_ show a significant negative correlation with various chemical property indicators. This indicated that as the *D*_1_ and *D*_1_/*D*_0_ increase, the corresponding chemical property indicators in the soil decrease. Furthermore, there is a strong correlation among the soil chemical property indicators, and total nitrogen and available potassium have higher correlation coefficients compared to other indicators (Table 3).

## Discussion

The hydrothermal conditions in the eastern mountainous Liaodong region are relatively good, however, with global chronic climate warming and uneven regional rainfall distribution, even in semi-humid areas, warming and reduced rainfall have led to an increase in tree mortality rate and decrease in forest coverage^41–44^. The deteriorating ecological environment poses a threat to the normal growth and development of trees, but it also promotes their gradual adaptation and improvement of the local environment^29^. For instance, the decomposition of forest litter can effectively improve soil aggregate structure, increase soil porosity and infiltration rate. The root systems of trees also play a role in soil improvement, significantly affecting soil moisture retention^45^. Therefore, different forest types have significant effects on forest soil^6,9,14,21,35^. Since forest soil carries the entire life of the forest ecosystem, the quality, condition, and properties of its internal structure directly influence the growth and survival of organisms above and below ground, providing the necessary material foundation for their growth and development^29,45^. Thus, studying changes in soil properties of typical forest types is necessary. Currently, with the help of fractal theory, especially the concept and application of fractal dimension in soil science research, related technologies, theories, and instruments are becoming mature^8,15^. The related research involves soil mechanical composition, soil moisture characteristic curve, soil structure, solute transport, and other fields^2^. In this study, fractal theory was used to compare and study the fractal dimension characteristics, physical and chemical differences, and the relationship between the fractal dimension distribution of soil particle size and the basic soil properties of typical forest types in the sub-humid area in Northern China. It aims to quantitatively describe the soil structure status of different forest soils, the relationship between forest types and soil physical and chemical properties, reveal the mechanism of their impact on soil physicochemical properties by comparing the differences in internal properties of forest soils of different types, and provide a scientific basis for preventing degradation, ecosystem restoration, and later management and reconstruction in the sub-humid area, and the same climate regions around the world.

Fractal analysis offers the opportunity of quantifying and integrating information on soil biological, chemical, and physical phenomena. A better approach is combining laser diffraction method and fractal analysis, which allows a better understanding of the performance of the soil system^46,47^. Recent studies showed the fractal dimensions of PSD increased with clay content but decreased with sand content following a linear trend^2,15^. These findings determined that the selected removal of clay and silt fractions resulted in decreasing D values^16^, and our research results are in agreement with the mentioned studies above. Moreover, vegetation solutions, not only natural but also artificial forests, also have significant effects on soil PSD and D values. Previous studies indicated that topsoil profile of natural grassland, woodland, arboreal forest, and shrub land could obviously decrease the soil erosion and increase the soil clay content, thus it usually had higher fractal dimensions of PSD due to the well covered vegetation^15^. Since clay and silt are more easily eroded by runoff than sand^17^. Once Sandy Land loses the protection of covered vegetation, or wind velocity and precipitation exceed the threshold, accumulative fine particles like clay and silt fractions can be rapidly eroded and lost. Overall, in our study, anti-desertification solutions like forests establishment had a considerable effect in the increase of the fine fractions, and soil PSD recovered more as broadleaf forests and mixed conifer-broadleaf forests establishments (Table S1), which support our first hypothesis.

Forest soil has a loose structure and contains more water-stable aggregates, making it the main source for water conservation^48–50^. The ability of forest land to distribute and regulate precipitation is reflected in the soil water infiltration rate, which is mainly attributed to the differences in capillary porosity and capillary moisture capacity among different soil types^15^. The main reason for these differences is related to the attributes and distribution of forest litter^51^. Generally, the decomposition rate of coniferous litter is relatively fast, leading to the formation of a large amount of high-quality humus, which contributes to a loose soil structure^51,52^. However, excessively high stand density can also affect stand structure, so it is necessary to choose an appropriate planting density^51,52^. The values of total capillary porosity and capillary porosity of the soil in this study are consistent with previous research results, indicating that the decomposition of litter and other processes have significantly improved soil structure, resulting in effective control of soil degradation after afforestation^53^.

Studies have shown that the saturation moisture capacity of forest soils, including *P*. *koraiensis*, *P*. *sylvestris*,* Q*. *mongolica*, *J*. *mandshurica*, and *P*. *koraiensis* × *Q*. *mongolica* forests is much higher than the CK (Fig. 6). In this study, the soil’s capillary porosity is relatively high, indicating that the litter decomposition rate in forest land is fast and the root system of the forest land is deep. The deep root system penetrates into the soil, significantly improving the internal structure of the soil. This leads to a more stable and permeable aggregate structure of the soil, reducing water flow resistance and increasing soil permeability. Meanwhile, moisture can be retained in the soil for a longer period, ensuring the soil’s water-holding capacity, this complementary hydrological effect helps restore local soil degradation and plays a role in conserving water resources^54^. Generally, the ability of forest land to distribute and regulate precipitation is reflected in the soil water infiltration rate, which is primarily due to the differences in capillary porosity and saturation moisture capacity among different soil types^55^. The decomposition rate of litter from coniferous tree species is relatively fast, leading to the formation of a large amount of high-quality humus and soil structure improvement^56^. In this study, the capillary porosity and saturation moisture capacity of *P*. *koraiensis* and *P*. *sylvestris* forests are slightly lower than those of *Q*. *mongolica* and *P*. *koraiensis* × *Q*. *mongolica* forests (Fig. 6). The main reason is that broadleaf forests have a thicker litter layer compared to coniferous forests, resulting in a higher accumulation of litter (Table S2 and S3). The decomposition of a large amount of litter increases the nutrient content of the soil, promoting root growth and the survival of soil microorganisms. This, in turn, leads to good vegetation growth and the increase in above-ground and below-ground biomass positively contributes to soil structure improvement^45^. This significant recovery of the physical properties of the soil was observed. However, there were no significant differences among the different forest types, indicating that the litter decomposition and other processes have significantly improved the soil structure.

The differences in the content of N, P, and K among different forest types are related to the general nutrient status of the soil. In general, the chemical properties of the soil in *P*. *koraiensis*, *P*. *sylvestris*, *J*. *mandshurica*, *Q*. *mongolica*, and *P*. *koraiensis* × *Q*. *mongolica* forests are better than those of the CK (Fig. 7). Regarding soil organic matter, the soil organic matter content in the topsoil layer is higher than that in the sub-topsoil layer, indicating significant microclimatic effects within the forest and faster litter decomposition rates. This ensures the maintenance of nutrient content such as N, P, and K in the topsoil. Furthermore, studies have shown that compared to natural forests under natural regeneration, the pH values of all soil layers in artificial forests tend to decrease. This is directly related to the organic acids produced during the decomposition process of litter^57–59^. Specifically, in artificial coniferous forests, there is a significant trend of soil acidification, which is directly related to the production of organic acids during litter decomposition. However, this study did not show a trend of soil acidification.

The difference in particle size composition of the soil under the forest is mainly due to the external forces (wind erosion and water erosion) that transport, influence, accumulate, and erode the surface soil material^15^. Fine soil particles are the most easily eroded due to their small volume and light weight, resulting in an increasing content of sand particles^7^. Planting artificial forests and existing natural forests can increase surface roughness, stabilize drifting sands^3^. Meanwhile, the litter under the forest canopy can effectively intercept precipitation, reduce throughfall, increase stemflow, promote water accumulation in the forest, and ultimately reduce soil runoff^26^. This prevents external forces from directly affecting the surface soil and has a certain preservation and protection effect on fine particles, while the soil also undergoes a certain process of refinement. The changes in mean particle size and skewness indicate the process of concentration changes in soil texture, while the changes in standard deviation and kurtosis represent the dispersion changes of coarse and fine soil particles. The changes in these two indicators show opposite states^57–59^. Fractal dimension is the most important parameter in fractal theory and as a comprehensive index to evaluate the degree of soil evolution- the fractal dimension value is better than various single indicators of soil texture parameters such as mean particle size, skewness, standard deviation, and kurtosis^60–62^. In this study, the changes of mean particle size, skewness, and fractal dimension with different treatments (*P*. *koraiensis*, *P*. *sylvestris*,* J*. *mandshurica*, *Q*. *mongolica*, and *P*. *koraiensis* × *Q*. *mongolica* forests and CK plots) showed certain regularity, while the changes in standard deviation and kurtosis were not significant (Fig. 3). In terms of singular fractal dimension values, the *P*. *koraiensis* × *Q*. *mongolica* forests had the highest D value, indicating that the structure of litter under this forest type is more complex, with a high degree of layering between needleleaf litter and broadleaf litter. Acidification of needleleaf litter in the decomposition process can accelerate the decomposition of broadleaf litter and branches, resulting in a thick layer of litter that directly decomposes and forms more humus on the soil surface. This improves the internal structure and physical and chemical properties of the soil, enhances permeability, infiltration capacity, and chemical content. The fractal parameters reflecting the geometric shape of soil structure show that the higher the clay content, the higher the fractal parameter, while the lower the sand content, the lower the fractal parameter. Regression analysis of soil particle size fractal parameters and particle content at various sizes shows that clay and silt content are positively correlated with soil particle fractal parameters, while sand content is negatively correlated^4,16,20^. The correlation coefficients vary depending on the parameters. In this study, the correlation coefficient between clay content and fractal parameters was the highest, followed by silt content and sand content, which indicates that although soil particle fractal parameters can reflect the composition or homogeneity of soil particles, they respond differently to the content of different size soil particles (Fig. 5). The results of this study also showed that the mean particle size and fractal dimension of soil layers under the forest are greater than those of CK, and they increase from pure tree species forests to mixed conifer-broadleaf forests. his indicates that the fine soil particles gradually recover under the long-term protection of forests, and the recovery effect of mixed conifer-broadleaf forests is significantly better than that of pure forests. The restoration of topsoil is faster than that of sub-topsoil layers, indicating that the restoration effect of vegetation is more significant on the topsoil layer. The results of this study are consistent with previous studies by other scholars^5,8,17,46^. The results showed that soil singular fractal dimension has a positive correlation with vegetation cover and an inverse correlation with wind erosion intensity. Moreover, multiple fractal parameters show a trend of *D*_0_ (capacity dimension) > *D*_2_ (correlation dimensionn) > *D*_1_ (information dimension), indicating that soil particle size distribution is not completely uniform, and it is necessary to perform multiple fractal analysis (Fig. 4), which support our second hypothesis.

Soil particle size and content of sand and clay are altered by the natural processes of soil transport and deposition in the environment, thereby influencing the fractal parameters of soil particles under different land use types^13,63^. Forested areas have a higher proportion of fine particles, resulting in significantly larger fractal dimensions compared to non-forested areas^59^ The geometric shape of the soil is reflected by its fractal characteristics, which directly affect the physical properties of the soil particle composition, as well as the uniformity of soil particle size and texture^58^. Therefore, using fractal parameters as comprehensive indicators to quantitatively describe soil properties holds great research value and potential^7,17,39^. The size of soil particle diameter plays a decisive role in the binding between soil particles, the size and quantity of pores, and their geometric morphology^64–66^. Fractal parameters of the soil are the parameters that reflect the geometric structure of the soil^67–70^. In general, higher clay content corresponds to higher fractal parameters, while lower sand content is associated with higher fractal parameters. In this study, through regression analysis of the single and multiple fractal parameters of soil particle size and the content of different particle sizes, it is found that the overall soil particle fractal parameters are positively correlated with clay and silt content, and negatively correlated with sand content. Meanwhile, the correlation coefficients vary depending on the specific parameters. In this study, the correlation between the fractal dimension of soil particle size and soil physicochemical properties was investigated. The results showed that soil fractal parameters are highly positively correlated with capillary porosity, and highly negatively correlated with soil bulk density, which is not consistent with the findings of previous studies^8,15,17,46^. This discrepancy may be due to the influence of human factors on the soil in practical applications, whereas the sampling sites selected in this study have minimal human disturbances. Therefore, the conclusions of this study differ from those of previous studies. It has been shown in previous research that fractal parameters of soil particle composition have great potential for application in soil fertility diagnosis indicators^70–72^.

This study selected 12 relatively independent variables to analyze the relationship between the fractal characteristics of soil particles and soil physicochemical properties, which comprehensively reflect their relationship. However, due to the limitations of sampling conditions, future research should focus on different slopes and slope positions to explore the impact of vegetation restoration on soil physicochemical properties under different site conditions. Additionally, other environmental factors such as salinity, relative humidity, accumulated temperature, radiation, atmospheric temperature, average wind speed, rainfall, precipitation intensity, soil moisture content, and soil thermal flux directly and significantly affect soil physicochemical properties. These factors may introduce certain biases into the experimental results. Furthermore, factors such as forest age and stand density should be considered in future research, as they have a significant impact on the soil improvement effects of different forest types. Therefore, further in-depth research is needed based on the relevant research results mentioned above.

Overall, the use of multi-fractal methods and spectral functions to describe complex bodies with fractal structures enables the study of all characteristics starting from a systemic perspective^59–62^. By employing statistical physics methods, the distribution patterns of complex bodies can be discussed^64^. This allows for the extraction of rich information regarding the spatial distribution of soil particles with fractal properties, which can be quantified^65^. Multi-fractal methods not only reveal the overall characteristics of the spatial distribution of soil particle percentages from dense to sparse, but also analyze the distribution characteristics of specific particle size percentages^67–69^. This overcomes the limitations of single-fractal methods, which can only describe and characterize the overall and average features of soil particle fractal properties^70–72^. The use of laser particle size analyzers overcomes the discrepancies caused by the segmentation of particle size ranges in the calculation of fractal dimensions and provides a more intuitive visualization of certain abnormal transformations in soil particle size ranges^65^. Multi-fractal methods can analyze the local variability and heterogeneity of soil structure, and using spectral functions to describe soil structures can incorporate more characteristic information than single-fractal dimensions^71^. Numerous scholars have used multi-fractal methods to study soil particle size distributions^68–70^. This study further demonstrates that multi-fractal analysis provides more information than single-fractal analysis and better represents the intrinsic structural features of the soil. Therefore, the use of multi-fractal analysis methods to study the soil in this area can fully describe the entire range of particle size distribution and provide a precise analytical method for quantitatively studying soil particle size distribution and its structural characteristics.

## Conclusions

To evaluate soil fractal characteristics and soil physicochemical properties of *Pinus koraiensis*, *Pinus sylvestris* var. *mongolia*, *Quercus mongolica*, *Juglans mandshurica* and mixed conifer-broadleaf (*Pinus koraiensis* × *Quercus mongolica*) forests in the sub-humid area of eastern Liaoning province in Northern China. The topsoil (0–20 cm) and sub-topsoil (20–40 cm) samples, and litter were collected simultaneously, and the relationship between the soil particle size characteristics and properties under natural cultivation measures were evaluated and compared as control check (CK). The following conclusions were inferred from this study:

1. The soil composition in the study area is mainly composed of sand content, followed by silt and clay contents. The overall particle size characteristics show well sorted, positive skewness, and narrow kurtosis shape. The mean particle size and singular fractal dimension of the soil under different forests are larger than those of the CK plots, indicating that the fine soil particles gradually recover under long-term protection of forest, and the recovery rate of the topsoil is greater than that of the sub-topsoil. The multiple fractal dimensions show a trend of *D*_0_ > *D*_2_ > *D*_1_, indicating that the particle size distribution of the soil is not an ideal type of uniform distribution, and the evaluation of soil quality using singular fractal dimension still needs to be combined with multiple fractal analysis.
2. The fractal dimensions and soil physiochemical properties in the understory of mixed conifer-broadleaf forests are higher than those of other forests and the CK. It is worth mentioning that, there is no significant difference in soil pH among the study plots, and there is no obvious acidification tendency, especially in *P. koraiensis* and *P*. *mongolia* stands.
3. Pearson correlation analysis shows that there is a positive correlation between physical and chemical indicators. The singular fractal dimension and capacity dimension are significantly positively correlated with various indicators, indicating that using fractal theory to assess the spatial distribution of soil physical and chemical nutrients is feasible in the study area.

The results showed that soil fractal characteristics could interpret the physicochemical properties of soil from the internal structural characteristics of soil, and then provide a powerful scientific basis for guiding the optimization of stand management in this area. Moreover, to probe into the comprehensive effect of vegetation on soil improvement and the difference among various forest types, and to find out whether the difference of soil properties has supporting effect on vegetation, so as to provide a reliable scientific treatment method for forestry management and reconstruction in the later stage. The highlights of this study are: i) The broadleaf and mixed conifer-broadleaf forests are suitable for local afforestation for soil degradation restoration purpose; and ii) It is necessary to use the singular fractal dimension to evaluate soil quality in combination with multifractal analysis. These two highlights of this study also support our first and second hypothesis.

The limitation of this study is by the omission of other soil depths and microelement levels. For instance, *Pinus sylvestris* var. *mongolia*s is a shallow-rooted plant and 80% of its roots are found at 0–100 cm soil depth. Other soil nutrients may have significant direct or indirect impact on plant growth and soil properties. Future studies should address these limitations.

## Supplementary Information

Supplementary Information.

## Data Availability

All data generated or analyzed during this study are included in this its supplementary information file (data.xls).
